# Successful Treatment of Posttransplant Refractory Pure Red Cell Aplasia Following Parvovirus B19 Infection

**DOI:** 10.1155/carm/5542026

**Published:** 2026-02-03

**Authors:** Yousef Ansara, Omar Marouf, Khalil Abualhumos, Mohammed AbuBaha, Hossam Salameh, Huda Saadeddin, Mohammad Al-Sheikh

**Affiliations:** ^1^ Faculty of Medicine, Al-Quds University, Jerusalem, State of Palestine, alquds.edu; ^2^ Department of Internal Medicine, Palestine Medical Complex, Ramallah, State of Palestine; ^3^ Department of Medicine, An-Najah National University, Nablus, State of Palestine, najah.edu; ^4^ Department of Nephrology, Palestine Medical Complex, Ramallah, State of Palestine

## Abstract

Pure red cell aplasia (PRCA), a rare cause of anemia limited to the erythroid lineage, is characterized by normocytic normochromic anemia, severe reticulocytopenia, and markedly reduced or absent erythroid precursors in the bone marrow. We report a 44‐year‐old male with end‐stage renal disease (ESRD) secondary to autosomal dominant polycystic kidney disease (ADPKD) who developed refractory PRCA following a live‐donor renal transplant. One month posttransplant, the patient presented with severe, persistent anemia accompanied by fatigue and dyspnea. Initial management included blood transfusions, vitamin B12 supplementation, and adjustments to immunosuppressive therapy due to suspected drug‐induced cytopenia. Bone marrow biopsy confirmed PRCA, and polymerase chain reaction (PCR) revealed a persistently elevated parvovirus B19 infection, a commonly recognized etiology of PRCA in immunocompromised patients. Treatment included intravenous immunoglobulin (IVIG) and frequent blood transfusions. Despite therapy, the patient experienced recurrent anemia, pancytopenia, and febrile neutropenia. Over successive hospitalizations, hematologic improvement was achieved with hemoglobin stabilization and significant viral load reduction. This case underscores the diagnostic and therapeutic complexity of managing parvovirus B19–induced PRCA in posttransplant patients, emphasizing the need for individualized strategies incorporating IVIG, supportive care, and tailored immunosuppressive regimens.

## 1. Introduction

Anemia following kidney transplantation is characterized by hemoglobin levels below 12 g/dL in women and 13 g/dL in men. It is a common complication with multiple potential causes, including deficiencies in iron and/or erythropoietin (EPO), perioperative blood loss, immunosuppressive therapies, and viral infections. Differential diagnoses must be thoroughly evaluated, often revealing multiple contributing factors, which can complicate the timely diagnosis of less common forms of anemia [1].

Common viral infections, including parvovirus B19, may ultimately cause significant acute and chronic anemia in immunocompromised individuals. Parvovirus B19 is a small virus, approximately 25 nm in diameter, with a single‐stranded DNA genome. It is frequently encountered in childhood, with over half of adolescents showing specific antibodies to the virus. Transmission primarily occurs through respiratory droplets, and nearly half of adults may experience asymptomatic infections [2]. When symptoms do occur, patients may present with flu‐like symptoms during the viremia phase, which can be followed by rashes and/or joint pain; children are more likely to develop rashes, while adults typically report joint discomfort. The virus can also be transmitted via blood, blood products, or transplanted organs [3].

Parvovirus B19 infects and replicates in erythroid progenitor cells in the bone marrow, leading to apoptosis of these cells. A key feature of parvovirus B19 infection is a decrease or absence of reticulocytes, even in immunocompetent individuals. Transient anemia and other cytopenias may also be observed. Importantly, parvovirus B19 can cause a transient aplastic crisis in individuals with underlying hemolytic disorders, such as hereditary spherocytosis or sickle cell disease, and can result in fetal loss or hydrops fetalis during pregnancy, as well as pure red cell aplasia (PRCA) in immunocompromised patients [4].

PRCA, a less common cause of anemia, is limited to the red cell lineage and is characterized by normocytic normochromic anemia, severe reticulocytopenia, and a marked reduction or absence of erythroid precursors in the bone marrow. Acquired PRCA can arise from lymphoproliferative disorders, autoimmune conditions, specific medications, viral infections (most notably parvovirus B19), thymoma, and other malignancies. Patients receiving immunosuppressive therapy are at an elevated risk for parvovirus B19–related PRCA due to sustained viremia and suppressed erythropoiesis [5].

Management of parvovirus B19–related complications in kidney transplant recipients typically includes blood transfusions, adjustments to immunosuppressive therapy, and intravenous immunoglobulin (IVIG) treatment [6].

This case report will discuss refractory parvovirus B19–induced PRCA in a 44‐year‐old male patient who underwent kidney transplantation.

## 2. Case Presentation

A 44‐year‐old male patient with a history of end‐stage renal disease (ESRD) due to autosomal dominant polycystic kidney disease (ADPKD) underwent bilateral nephrectomy without any complications. After 8 months of hemodialysis (HD), he was a good candidate for renal transplantation and received a kidney from his nephew, who was a *Cytomegalovirus* (CMV) seronegative, cross‐matched, HLA‐matched live donor with a panel‐reactive antibody (PRA) negative.

Following the kidney transplant operation, he was hospitalized and given anti‐thymocyte globulin (ATG) induction, with no postoperative complications. After transplantation, the graft was functioning immediately, and the kidney function status was normalized. He was subsequently discharged and maintained on triple immunosuppressive medications, including mycophenolate mofetil (MMF) 360 mg twice daily, tacrolimus 5 mg twice daily, and prednisolone 30 mg once daily with rapid tapering, in addition to prophylactic antibiotics and antivirals, including trimethoprim–sulfamethoxazole 800/160 mg twice daily and valganciclovir 450 mg twice daily.

Other comorbidities include hypertension of 10 years duration, maintained on amlodipine 5 mg and carvedilol 12.5 mg.

During routine follow‐up with his nephrologist 1 month after transplantation, he started complaining of generalized weakness, fatigue, inability to perform daily activities, exertional shortness of breath on minimal activity, and orthostatic dizziness. He also noticed that his skin had become pale. On review of systems, he did not have any other complaints. Laboratory analysis was done and showed anemia with a hemoglobin level of 5.6 g/dL and mean corpuscular volume of 90.1 fL. He was referred to the hospital and was admitted to the internal medicine ward for investigation of anemia and blood transfusion.

Physical examination showed a vitally stable man, with a body mass index (BMI) of 28 kg/m^2^. Physical findings were consistent with conjunctival pallor and hyperdynamic precordium; otherwise, it was unremarkable.

Throughout his hospitalization, further laboratory workup was done, as depicted in Table 1. The results were consistent with normocytic anemia, with no evidence of hemolysis, and normal platelet and white blood cell (WBC) counts. Peripheral blood smear was done and showed microcytic hypochromic red blood cells (RBCs) with erythroblasts and no signs of hemolysis. Although the patient’s MCV values were within the normal range, the smear demonstrated microcytic and hypochromic morphology. This discrepancy likely reflects iron‐restricted erythropoiesis and inflammation, which can produce morphologic microcytosis despite preserved mean corpuscular volume. Megaloblastic changes with hypersegmented neutrophils were detected. Platelets were normal in shape and number. Reticulocyte count was 0.2%. Accordingly, the patient received 2 units of leukocyte‐depleted packed RBCs (PRBCs). The iron profile was not informative, since the patient was already on a multiple‐drug regimen, as demonstrated in Table 1. Polyoma BK virus and CMV panels were negative. Vitamin B12 levels were severely low; therefore, he was suspected to have a combined cause of anemia due to an inflammatory process, iron deficiency, and vitamin B12 deficiency. Accordingly, he was managed with intramuscular (IM) injections with cyanocobalamin of a dose of 30 mcg daily for a duration of 6 days, along with folic acid 5 mg once daily, and was given EPO as a line on management. He was subsequently discharged after an increase in hemoglobin level and was recommended to follow up in the outpatient clinic (OPC) after 1 week.

**TABLE 1:** Laboratory values throughout the disease course.

Lab test	First presentation	Second presentation	Third presentation	Fourth presentation	Fifth presentation	Sixth presentation	Seventh presentation
CBC							
Hemoglobin (g/dL)	5.6	4.7	5.5	4.6	7.6	7.5	5.3
MCV (fL)	90.7	88.9	84	87	84.7	84.2	87
WBC (10^3^/μL)	4.1	1.8	2.6	2.3	4.1	1.6	3.5
Granulocytes (%)	90.2	56	69.4	72.9	88.7	71.7	59.5
Lymphocytes (%)	8.3	17.7	16.4	11.9	3.8	15.2	11.1
Platelets (10^3^/μL)	242	227	294	134	193	63	9
Red blood cells (× 10^9^/L)	1.82	1.62	1.94	1.66	2.09	2.66	1.77
Reticulocyte count	0.2%	—	—	—	—	—	—
CRP	1.4	1.1	1	1.5	6	2.3	3.1
ESR	90	35	97	40	35	—	140
Iron profile							
Total serum iron (mcg/dL)	246	249	220	238	—	216	272
Ferritin (μg/L)	1044	1470	2665	1675	—	7572	4964
Transferrin (g/L)	172	—	—	—	—	156	198
Total iron binding capacity (μmol/L)	—	—	< 250	—	—	—	—
Vitamin B12 level (pg/mL)	< 148	—	2422	> 2000	—	—	1175
Serum electrolytes							
Sodium (mEq/L)	135	137	131.9	134	129	129.4	131.3
Potassium (mEq/L)	5.10	4.90	5.4	4.7	4.7	4.9	4.9
Calcium (mg/dL)	8.48	8.71	8.92	—	—	8.2	8.33
Kidney function							
Creatinine (mg/dL)	1.19	1.77	1.64	1.29	1.40	1.25	1.36
BUN (mg/dL)	27.02	28.9	25.38	21.42	26.90	22.6	39.3
Tacrolimus level (ng/mL)	10.2	8.2	14.2	—	—	—	—
Stool for occult blood	Negative	—	—	—	—	—	—
Lactate dehydrogenase (LDH) (IU/L)	173	248	—	243	—	333	219
Total bilirubin (mg/dL)	0.31	0.36	—	0.6	—	1.04	1.03
Direct bilirubin (mg/dL)	0.115	0.14	—	0.2	—	0.5	0.478

*Note:* The table demonstrates the relevant laboratory data collected over a course of seven hospitalizations for the patient presented in this case.

**FIGURE 1:**
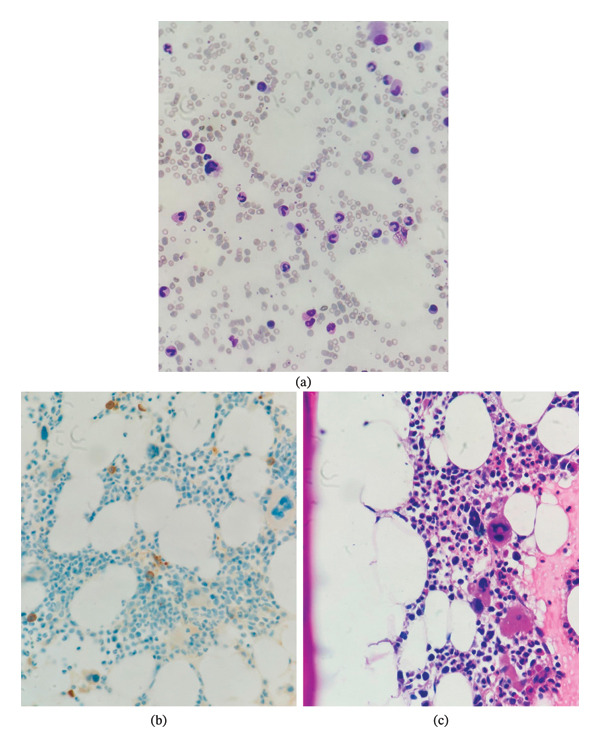
This figure shows the results of the bone marrow biopsy (500x magnification), as shown using Wright–Giemsa stain (a). There is a significant reduction in erythroid cells, with no definite erythroid colonies identified on both immunohistochemical CD71 (100x magnification) (b) stain and the hematoxylin and eosin (H&E) stain (100x magnification) (c), except for a few scattered erythroid precursors. The bone marrow aspirate also demonstrates a marked reduction in erythroid cells, with no identifiable erythroid precursors observed.

On follow‐up with the nephrologist, laboratory analysis was repeated since the patient still experienced signs and symptoms of anemia. Results showed a further drop in hemoglobin levels to a value of 4.7 g/dL. Other labs were similar to his previous admission, as demonstrated in Table 1. Therefore, he was admitted once again for blood transfusion and investigation of anemia. MMF was discontinued due to suspected drug‐induced hematologic cytopenia. He received 3 units of leukocyte‐depleted PRBCs, and his hemoglobin level increased to 7.8 g/dL. An abdominal ultrasound was done to rule out organomegaly, which showed a normal transplanted kidney. The liver showed multiple variable‐sized, benign‐looking cysts. Ultrasound was otherwise unremarkable. Due to the findings mentioned and possible EPO‐induced aplastic anemia, the nephrologist referred the patient to a hematologist, and he was sent for a bone marrow biopsy, as shown in Figure 1. As the report shows, bone marrow was 50% normocellular for age. Erythroid cells were significantly decreased, with no definite erythroid colonies identified on H&E stain.

Cellularity was appropriate for age (∼50%), with preserved myeloid and megakaryocytic lineages. No giant proerythroblasts or intranuclear viral inclusion bodies were identified. No typical intranuclear inclusion bodies (giant pronormoblasts with “lantern” or dark‐stained inclusions) were observed in our bone marrow aspirate. CD71 highlights scattered erythroid precursors. Myeloid, megakaryocytes, lymphocytes, and plasma cells were visualized normally. These findings are consistent with PRCA.

A polymerase chain reaction (PCR) quantitative assay for parvovirus B19 was done, showing a result of 6.93 × 10^7^ copies/mL. With bone marrow biopsy findings and a positive HPV‐B19 PCR, the clinical picture was consistent with HPV‐B19–induced PRCA. Accordingly, he was given 4 doses of 0.4 g/kg/day for 4 consecutive days of IVIG according to current institutional management protocol, and afterward, he was discharged for follow‐up after 1 week to evaluate the response. Quantitative parvovirus B19 PCR was performed on plasma using a real‐time PCR assay reported in copies/mL, with a lower limit of detection not reported by the laboratory.

After 1 week, he presented to the nephrology OPC for follow‐up. Repeat CBC results showed a significant drop in his HGB to a level of 4.9 g/dL, and he still experienced symptoms of anemia. Parvovirus B19 quantitative assay follow‐up result showed a further significant increase in viral load to a level of 2.32 × 10^11^ copies/mL. Therefore, the patient was admitted to the internal medicine department once again for blood transfusion and received 2 units of leukocyte‐depleted PRBCs. He also received another cycle of IVIG similar to the previous protocol. Repeat CBC before discharge showed an increase in HGB level to 6.8 g/dL. His immunosuppressive medications were adjusted by changing MMF to cyclosporine 200 mg twice daily and by changing tacrolimus to everolimus 0.75 mg twice daily. Anti‐EPO antibody testing was not available at our institution and therefore was not performed. This is acknowledged as a limitation, and empiric discontinuation of ESAs was based on clinical suspicion. He was also given a novel class of EPO drugs called hypoxia‐inducible factor (HIF) inhibitor—vadadustat—due to a possible EPO‐induced aplastic anemia. He was then discharged on his drug regimen and recommended follow‐up after 1 week.

After immunosuppressive therapy was modified in a stepwise manner in response to persistent parvovirus B19 viremia and recurrent cytopenias, MMF was discontinued during the second hospitalization due to suspected drug‐induced cytopenia. Tacrolimus was subsequently discontinued during the next admission, and everolimus was initiated on the same day at 0.75 mg twice daily, targeting a trough level of 3–8 ng/mL, as per institutional protocol for mTOR‐based conversion. Cyclosporine was introduced at 200 mg twice daily with a goal trough level of 75–150 ng/mL. Dual cyclosporine–everolimus therapy was used only temporarily during the transition phase until therapeutic everolimus levels were achieved, after which cyclosporine dosing was tapered according to hematologic and virologic response. Kidney graft status was monitored clinically and biochemically throughout these adjustments, and no signs of rejection were observed. No biopsy was required, as serum creatinine remained stable and there were no clinical indicators suggestive of allograft dysfunction.

The patient’s clinical course was further complicated, as he was subsequently admitted three more times to the internal medicine ward due to symptomatic anemia and low Hb levels requiring blood transfusions and persistently high parvovirus B19 viral load. This prompted the administration of IVIG during each hospitalization with a similar treatment regimen.

His situation was complicated during one of these admissions by myelosuppression affecting all 3 lines. He became febrile, requiring a full septic workup, and was given empiric treatment with piperacillin–tazobactam. Blood culture results revealed bacteremia due to methicillin‐resistant *Staphylococcus aureus* (MRSA), and he received treatment with vancomycin. At the time of his neutropenic septic episode, the patient’s absolute neutrophil count (ANC) was markedly reduced. Based on Table 1 laboratory values, the ANC nadir was approximately 1.0 × 10^3^/μL (WBC 1.8 × 10^3^/μL with 56% granulocytes). Filgrastim 300 mcg subcutaneously daily was administered, after which ANC increased to above 2.0 × 10^3^/μL, paralleling clinical improvement and resolution of fever. This documented rise in ANC supports a therapeutic response to filgrastim during the episode of MRSA bacteremia. Given the known myelosuppressive effects of trimethoprim–sulfamethoxazole and valganciclovir in kidney transplant recipients, both medications were reviewed for renal dosing appropriateness. They were temporarily withheld during the episode of pancytopenia. Their contribution to the severity of the cytopenias was considered clinically significant, especially in the context of ongoing viral infection and intensive immunosuppression. After consulting with a hematology specialist, it was recommended to give the patient 300 mcg of filgrastim daily as subcutaneous (SC) injections. Repeat serum levels of vitamin B12 revealed a response to treatment (Table 1). Additional investigations were performed to exclude alternative etiologies of PRCA, including direct antiglobulin test, haptoglobin, thyroid function tests, hepatitis B and C serologies, HIV testing, and EBV serology. All were unremarkable. Copper level was not measured and is acknowledged as a limitation. After improvement of his clinical and laboratory status, he was discharged to be followed up by his nephrologist.

Filgrastim was administered exclusively to treat severe neutropenia associated with MRSA bacteremia and not as therapy for PRCA, as it does not stimulate erythroid precursors.

On outpatient follow‐up, he described resolution of his symptoms. On physical examination, he appeared well, and there were no clinically significant exam findings. His labs showed stabilization of his HGB to around a level of 14 g/dL on serial hemoglobin readings. Repeat parvovirus PCR showed a significant decrease in viral load to a level of 5.7 × 10^1^, which indicates clearance of the infection.

Follow‐up PCR demonstrated virologic clearance with a reduction to 5.7 × 10^1^ copies. Serial CBCs showed sustained hemoglobin stability (≈14 g/dL) with clinical improvement. Reticulocyte recovery paralleled hemoglobin stabilization.

## 3. Discussion

PRCA is a hematologic disorder that results from failure of erythropoiesis. It presents with a normocytic, normochromic anemia with reticulocytopenia, and an otherwise unaffected leukocyte and platelet counts and morphology. On bone marrow aspirate, PRCA is characterized by a decreased level or complete absence of erythroblasts. PRCA could be inherited, such as in Diamond–Blackfan syndrome, or acquired by means of several factors, including autoimmune disorders, neoplasms, lymphoproliferative disorders, drugs, and bacterial or viral infections [5].

Parvovirus B19 infection is an important cause of transient aplastic crisis, particularly in patients with underlying hematologic disorders such as hereditary spherocytosis or sickle cell anemia [5]. Studies have shown a high affinity for the virus to target erythroid progenitor cells, thus inhibiting erythropoiesis through targeted cell damage. Healthy individuals can clear the virus through IgG antibodies, thereby only causing an asymptomatic, transient aplastic anemia [5, 7]. On the other hand, people with hematologic disorders, who have a higher RBC turnover rate, will more likely experience symptomatic aplastic anemia.

Immunosuppressed individuals may experience PRCA due to parvovirus B19 infection, which could cause a persistent PRCA due to an inability to host an immune response against the virus [8]. Immunosuppressive agents such as tacrolimus, azathioprine, and MMF may cause drug‐induced PRCA [9]. In rare situations, people who undergo solid organ transplantation may develop PRCA due to their increased risk of contracting a parvovirus B19 infection, along with their exposure to immunosuppressive medications [9].

Throughout this report, we presented a case of a 44‐year‐old man with a known history of ADPKD. His disease course was complicated by ESRD, which required kidney transplantation. He received a kidney transplant from a live donor. Routine workup following his transplantation revealed the presence of anemia requiring blood transfusion, which was investigated to be of multifactorial causes, including iron deficiency, vitamin B12 deficiency, and drug‐induced cytopenia from MMF. Despite blood transfusion, management of his vitamin B12 deficiency, and discontinuation of the offending drug, he still had persistent anemia. Further investigation by bone marrow biopsy revealed findings consistent with PRCA. A PCR for parvovirus B19 showed a high viral load, which confirms the infection as the cause of PRCA.

Several case reports in the literature described post‐solid organ transplant PRCA due to parvovirus B19. The primary modality of treatment in the reviewed cases was based on alternation in the immunosuppressive regimen. In our case, we approached this entity by switching from MMF to cyclosporine and transitioning from tacrolimus to everolimus, which is supported by studies indicating that rapamycin inhibitors (mTORi), including sirolimus and everolimus, offer a viable alternative for immunosuppression in the transplantation setting [10]. Research has demonstrated that mTORi, unlike calcineurin inhibitors (CNIs) or MMF, may possess both immunosuppressive and antineoplastic properties [11]. Consequently, the early initiation of mTORi has significant clinical implications for managing transplant patients, particularly in achieving lower CNI levels [11].

Regarding renal function, mTORi are recognized for their favorable renal profiles. Most studies endorse the principle of “the sooner, the better,” suggesting that initiating mTORi promptly can prevent severe deterioration in glomerular filtration rate (GFR) [12]. Early initiation of mTORi, alongside a reduced dose of CNIs, has been linked to improved stabilization or enhancement of renal function. Therefore, a low dose of everolimus, as an mTOR inhibitor, has been incorporated into the treatment regimen, considering the high risk of rejection and the antiviral potential of mTOR inhibitors [13].

Measures to reverse PRCA, possibly due to anti‐EPO antibodies, may include the use of novel class drugs, including HIF drugs such as vadadustat or roxadustat. These drugs act by increasing endogenous EPO production, which has been implemented in the treatment of anemia due to CKD [14]. Many case reports described the use of HIF, which improved reticulocyte and hemoglobin concentration following administration, decreasing the need for blood transfusion [14]. This approach was attempted in our patient, following the suspicion of EPO‐induced PRCA.

A case reporting 2 patients in the literature showed that alteration in the immunosuppressive medication regimen was sufficient to recover the erythroid function in one patient [15]. However, the other patient required additional therapy by using 4 doses of IVIG to achieve complete remission of the disease [15]. Numerous cases described the use of IVIG for the treatment of parvovirus B19–related PRCA. IVIG contains anti–PV‐B19 IgG, which, over time, has remained the mainstay of treatment of anemia due to chronic PV‐B19 in immunocompromised individuals [16]. A retrospective study of 133 patients with parvovirus B19–related PRCA demonstrated a response to IVIG treatment in 93% of the cases only after the first dose. 33.9% of the described cases showed a refractory course of the disease [16].

Ersal et al. reported two kidney transplant recipients with relapsing parvovirus B19–associated PRCA, in whom giant pronormoblasts with dark‐stained intranuclear inclusion bodies were documented on bone marrow biopsy. After interruption/reduction of immunosuppression and repeated IVIG, the patients achieved hematologic remission, but both experienced relapse [17].

Refractory courses of the disease have been described quite rarely in the literature. In a course similar to the patient described in our case, a 49‐year‐old female patient who is a kidney transplant recipient was diagnosed with posttransplant PRCA due to a parvovirus B19 infection. Despite changing her medication regimen and receiving IVIG, she still had a persistently high viral load, for which she received further treatment with IVIG [18]. Another case described refractory PRCA due to HPV‐B19 following a pancreatic transplantation. The patient described in the report experienced a refractory course of blood transfusion requiring anemia, for which he later received courses of IVIG treatment on two occasions [19].

Management of persistent or hard‐to‐eradicate parvovirus B19 PRCA increasingly relies on repeated courses of IVIG, strategic reduction of immunosuppressive intensity, and transition to mTOR‐based regimens, which may decrease viral replication while maintaining graft protection. Emerging reports suggest that sequential IVIG dosing, maintaining trough IgG levels, and close monitoring of viral kinetics can be successful in resistant cases [19].

This case adds to the limited number of reported refractory courses where prolonged viremia persisted despite standard IVIG therapy. The sustained viral load > 10^11^ copies over multiple admissions, combined with recovery only after sequential IVIG cycles and modification to an mTOR‐based immunosuppressive regimen, offers insight into management strategies for extreme, prolonged cases.

## Funding

No funding was received for this manuscript.

## Ethics Statement

Written informed consent for publication of this case report and accompanying images was obtained from the patient. All identifying information has been removed to protect patient privacy. According to institutional policy, single‐patient case reports are exempt from formal IRB review.

## Conflicts of Interest

The authors declare no conflicts of interest.

## Data Availability

All data supporting the findings of this report are included within the manuscript.
